# Long-Term Outcomes of Cementless Bipolar Hemiarthroplasty in Young Patients With Osteonecrosis of the Femoral Head: The Impact of Implant Improvements and Preoperative Stage

**DOI:** 10.7759/cureus.57633

**Published:** 2024-04-04

**Authors:** Masanori Nishi, Yasushi Yoshikawa, Ichiro Okano, Yasutaka Kaji, Yuki Usui, Satoshi Okamoto, Shota Nakamura, Yoshifumi Kudo, Masanori Nakamura, Hideyo Miyaoka

**Affiliations:** 1 Orthopaedic Surgery, Showa University School of Medicine, Tokyo, JPN

**Keywords:** arthoplasty, young patients, long-term result, bipolar hemiarthroplasty, osteonecrosis of the femoral head

## Abstract

Introduction

Advancements in bipolar hemiarthroplasty (BHA) implants in the mid-1990s contributed to favorable short-term outcomes for osteonecrosis of the femoral head (ONFH), particularly in cases without acetabular cartilage lesions. Nevertheless, long-term results remain unclear. In this study, we investigated (i) the impact of new-generation BHA implants and (ii) the effect of the preoperative stage on long-term outcomes in young patients with ONFH.

Methods

The records of consecutive patients with ONFH who underwent cementless BHA were retrospectively reviewed. Patients aged ≥60 years, with <10 years of follow-up, or who underwent acetabular reaming during surgery were excluded. Radiographical and clinical outcomes of patients who received first-generation BHAs and new-generation BHAs (developed after 1998) were compared by stratifying based on preoperative stage 2/3A and 3B/4, according to the Japanese Investigation Committee classification.

Results

Overall, 50 hips from 39 patients (mean age: 44.6 years; 64% male) with an average follow-up of 18.6 years were included. The frequency of advanced-stage patients was significantly higher in the first-generation BHA group than in the new-generation group. Regarding postoperative outcomes, the first-generation BHA group had higher acetabular erosion grades (p<0.001) and more femoral component loosening than those in the new-generation group (p<0.001). Revisions were performed in eight hips (seven in the first-generation and one in the new-generation BHA groups, p<0.001). In the new-generation BHA group, there were no significant differences in patient background between stage 2/3A and 3B/4 groups, and only one case in the stage 3B/4 group required revision. In the new-generation group, the grade of acetabular erosion was significantly higher for stage 3B/4 than stage 2/3A (p<0.001); other radiographical and clinical outcomes did not differ significantly between stages.

Conclusion

New-generation BHAs have significantly better implant survival rates for early-stage ONFH than those of first-generation BHAs. These findings indicate that BHA is an acceptable treatment option for early-stage ONFH in young patients.

## Introduction

Osteonecrosis of the femoral head (ONFH) is a pathological condition that affects from young to older ages, resulting from compromised blood supply to the femoral head due to various etiological factors such as steroid use, alcoholism, and trauma [1]. In young patients, joint-preserving procedures such as core decompression, cell therapy, and femoral osteotomy may be considered [1-3]. However, in cases of extensive ONFH or femoral head collapse, arthroplasty is performed due to the ineffectiveness of other treatments [4]. Although total hip arthroplasty (THA) is usually considered the standard treatment option for extensive ONFH in young patients, bipolar hemiarthroplasty (BHA) could be an option for patients with ONFH without acetabular cartilage change [5]. BHA offers advantages over THA, including a lower incidence of dislocation, preserving the acetabular bone stock, shorter operative times, lower blood loss, and improved range of motion; however, early studies have reported poor outcomes of BHA for ONFH [6]. Over the last two decades, newly designed BHAs have replaced first-generation BHAs, such as the Bateman prosthesis. New-generation BHAs, which were initially introduced in the mid-1990s, are characterized by expanded oscillation angles, improved neck designs, porous coating of the femoral component, and installed locking rings [7-9]. Several studies have supported the benefits of these new-generation BHA implants in ONFH cases without acetabular involvement [10].

However, most reports on the new-generation BHA implants for ONFH cases have focused on mid-term outcomes, and long-term outcomes have not yet been determined. Therefore, this study had two aims: (i) to assess the impact of new-generation BHA implants on long-term outcomes of BHA for ONFH in young patients and (ii) to investigate the effect of the preoperative ONFH stage on long-term outcomes of new-generation BHA implants for ONFH in young patients.

## Materials and methods

Patients and data collection

This retrospective study was approved by the IRB of the authors’ affiliated institutions. The need for informed consent from each patient was waived owing to the study's retrospective nature.

The records of consecutive patients with ONFH who underwent cementless BHA between April 1984 and August 2008 at our hospital were retrospectively reviewed. New-generation BHAs, with various improvements [7-9,11], became available in 1998 at our institution. ONFH was diagnosed based on clinical history, physical examination, and imaging findings on radiographs and magnetic resonance imaging studies. Patients were categorized into two groups: those who underwent BHA with 1st-generation implants and those who underwent BHA with new-generation implants. Patients in the new-generation BHA group were also classified into stages 2/3A and 3B/4, according to the Japanese Investigation Committee (JIC) classification [12]. Stages of the JIC classification are as follows: stage 1, normal hip; stage 2, demarcating sclerosis without femoral head collapse; stage 3A, femoral head collapse <3 mm, including the crescent sign, without joint space narrowing; stage 3B, femoral head collapse ≥3 mm; and stage 4, secondary osteoarthritis.

Indication and surgical technique

In our treatment protocol for ONFH in young patients, BHA was only performed in patients who opted out of joint preservation procedures owing to extensive necrosis, social background, and preferences. All surgeries were performed by senior hip surgeons (H.M. and M.N.) using the posterior approach.

Radiological and clinical assessment

The radiographic analysis included the evaluation of bipolar head migration, subsidence of the femoral component, and osteolysis in the acetabulum and/or femur. The proximal migration of the bipolar head was assessed using the acetabular erosion grading system introduced by Baker et al. [13]. This system classifies the radiographic progression of bone erosion on the anterior-posterior (AP) radiograph into four grades: grade 0, no erosion; grade 1, narrowing of the articular cartilage, no bone erosion; grade 2, acetabular bone erosion and early migration; and grade 3, protrusio acetabuli. Subsidence of the femoral component was assessed by measuring the distance from the tip of the greater trochanter to the femoral component on the AP hip radiograph. Loosening of the cementless femoral component was defined as progressive subsidence ≥5 mm, based on the work of Callaghan et al. [14]. A board-certified orthopedic surgeon performed all radiographic evaluations. Immediate postoperative radiographs and those at the final follow-up were compared. Implant survival was investigated using the Kaplan-Meier method, with revision surgery as the endpoint. The clinical assessment utilized the visual analog scale (VAS) of the hip pain and the Merle d’Aubigné-Postel Score at the final examination, excluding revision cases.

Statistical analysis

Patient characteristics are described using frequencies and percentages, means and standard deviations, or medians and interquartile ranges, as appropriate. Chi-squared and Fisher's exact tests and the Mann-Whitney U test were used to evaluate binary and continuous variables, respectively. Statistical significance was set at p<0.05 for two-sided tests. All statistical analyses were performed using JMP Pro 17 (SAS Institute, Cary, NC, USA).

## Results

 

In total, 95 cementless BHA surgeries were performed on 80 patients during the study period. Of these cases, we excluded patients with the following conditions: (i) age at surgery >60 years (n = 27), (ii) follow-up <10 years (n = 13; failed follow-ups: 7, deaths: 4, and followed-up at an outside institution: 2), (iii) cases who underwent acetabular reaming during surgery (n = 4), and (ⅳ) missing data (n = 1). A total of 50 hips from 39 patients were included in the final analysis. Patient demographic characteristics are presented in Table 1.

**Table 1:** Patient demographics SD, standard deviation; AML A Plus, Anatomic Medullary Locking A Plus; BHA, bipolar hemiarthroplasty; MMA, Methyl Methacrylate; ONFH, osteonecrosis of the femoral head

Group	Total, n=50	1st-generation BHA, n=16	New-generation BHA, n=34	P-value
Age [years] Mean±SD	44.6 ± 9.3	43.6 ± 8.5	45.0 ± 9.8	0.459
Female n (%)	18 (36)	6 (38)	12 (35)	0.983
Follow-up [years] Mean±SD	18.6 ± 6.0	21.6 ± 7.7	17.2 ± 4.4	0.042
Cause of ONFH n (%)				
Corticosteroid	25 (50)	7 (44)	18 (53)	0.699
Alcohol	14 (28)	5 (31)	9 (26)	
Idiopathic	7 (14)	1 (6)	6 (18)	
Trauma	4 (8)	3 (19)	1 (3)	
Type C2 n (%)	41 (82)	14 (88)	27 (79)	0.699
Stage 2 n (%)	8 (16)	1 (6)	7 (21)	0.027
Stage 3A n (%)	9 (18)	0 (0)	9 (26)	
Stage 3B n (%)	28 (56)	13 (81)	15 (44)	
Stage 4 n (%)	5 (10)	2 (13)	3 (9)	
Side [Left] n (%)	24 (48)	7 (44)	17 (50)	0.767
Outer head size [mm] Mean±SD	47.9 ± 3.7	47.1 ± 2.3	48.2 ± 4.2	0.363
Femoral component	-	Harris-Galante: 8	Spongiosa Metal: 26	-
		Bateman: 6	Perfix: 4	
		MultiLock: 1	AML A Plus: 3	
		MMA: 1	Synergy Select: 1	

The first-generation BHAs used during the study period were Harris-Galante (Zimmer, Warsaw, IN, USA; n = 8), Bateman Universal Proximal Femoral (3M, St Paul, MN, USA; n = 6), MultiLock (Zimmer; n = 1), and Methyl Methacrylate (Kyocera, Osaka, Japan; n = 1). The new-generation BHAs used were Spongiosa Metal (ESKA Implants, Lübeck, Germany; n = 26), Perfix (Kyocera; n = 4), Anatomic Medullary Locking A Plus (Depuy Synthes, Warsaw, IN, USA; n = 3), and Synergy Select (Smith & Nephew, Memphis, TN, USA; n = 1).

The causes of ONFH were corticosteroid use (n = 25), alcohol consumption (n = 14), idiopathic (n = 7), and trauma (n = 4). The preoperative status of the hip joints was determined according to the JIC classification [9]: nine hips were type C1 and 41 hips were type C2. Eight hips were classified as stage 2, nine as stage 3A, 28 as stage 3B, and five as stage 4.

The preoperative stage was significantly more advanced in the first-generation BHA group than in the new-generation BHA group (p=0.027). There were no significant differences in other parameters between the first-generation BHA and new-generation BHA groups. Regarding major perioperative complications, there was no periprosthetic joint infection in this series. One patient in the new-generation BHA group experienced an intraoperative periprosthetic fracture; however, no additional procedure was required. There was no femoral component subsidence case in the series. Dislocation occurred early postoperatively in one case in the new-generation BHA group but did not result in repeated dislocation. A comparison of first and new-generation BHA groups is presented in Table 2.

**Table 2:** Comparison of radiological outcomes for first-generation BHA and new-generation BHA BHA, bipolar hemiarthroplasty

Group	Total, n=50	1st-generation BHA, n=16	New-generation BHA, n=34	P-value
Acetabular erosion grade n (%)				
0	16 (32)	0 (0)	16 (47)	<0.001
1	20 (40)	5 (31)	15 (44)	
2	7 (14)	5 (31)	2 (6)	
3	7 (14)	6 (38)	1 (3)	
Femoral component loosening n (%)	10 (20)	9 (56)	1 (3)	<0.001
Revision n (%)				
Total number of revision cases at 10 years after surgery	1 (2)	1 (6)	0 (0)	0.320
at 15 years after surgery	4 (8)	3 (19)	1 (3)	0.091
at 20 years after surgery	8 (16)	7 (44)	1 (3)	<0.001

**Figure 1:**
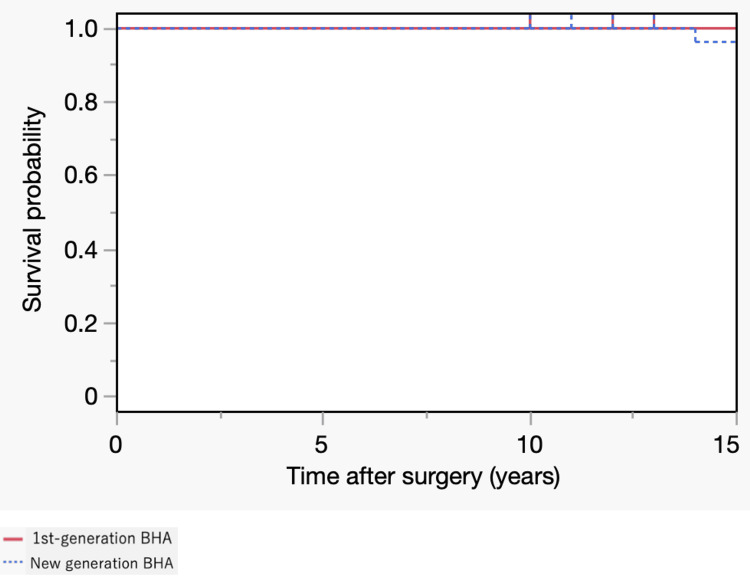
Kaplan–Meier Survival Curves with the endpoint of revision surgery (first-generation bipolar hemiarthroplasty (BHA) vs. modern BHA).

The distribution of the grade of acetabular erosion at the final follow-up, according to Baker’s classification, revealed significant differences between the two groups (p<0.001). Moreover, femoral component loosening was significantly more frequent in the first-generation BHA group than in the new-generation BHA group (p<0.001). At the time of the most recent follow-up, revisions had been performed in eight (16%) hips of eight patients (seven in the first-generation BHA and one case in the new-generation BHA group) (Figure 1).

Revisions were performed at an average of 14.7 (range: 10-18) years postoperatively, and all cases were converted to THA. At the 20-year postoperative mark, the revision rate was significantly higher for the first-generation BHA group than the new-generation BHA group, as indicated in Table 2; there were seven cases of censoring in the first-generation BHA group and one case in the new-generation BHA group. Regarding the differences in disease stages, the patient characteristics and results for the stage 2/3A and stage 3B/4 groups treated with new-generation BHAs are presented in Table 3.

**Table 3:** The comparisons of patients with new-generation BHAs divided into two groups by preoperative stage SD, standard deviation; ONFH, osteonecrosis of the femoral head; AML A Plus, Anatomic Medullary Locking A Plus; MMA, Methyl Methacrylate; VAS, visual analog scale

Group	Total, n=34	Stage 2/3A, n=16	Stage 3B/4, n=18	P-value
Age [years] Mean ± SD	45.6 ± 10.2	47.3 ± 9.6	43.0 ± 9.8	0.133
Female n (%)	13 (37)	4 (25)	9 (47)	0.173
Follow-up [years] Mean ± SD	17.2 ± 4.4	16.3 ± 4.1	18.1 ± 4.5	0.258
Cause of ONFH n (%)				
Corticosteroid	19 (54)	10 (63)	9 (47)	0.542
Alcohol	9 (26)	3 (19)	6 (32)	
Idiopathic	6 (17)	2 (12)	4 (21)	
Trauma	1 (3)	1 (6)	0 (0)	
Type C2 n (%)	28 (80)	10 (63)	18 (95)	0.032
Side [Left] n (%)	17 (49)	7 (44)	10 (53)	0.601
Outer head size [mm] Mean ± SD	48.3 ± 4.2	49.7 ± 3.3	46.9 ± 4.6	0.068
Femoral component				
Spongiosa Metal	26	12	14	0.476
Perfix	4	3	1	
AML A Plus	3	1	2	
Synergy Select	1	0	1	
Acetabular erosion grade n (%)				
0	16 (47)	13 (81)	3 (17)	0.001
1	15 (44)	3 (19)	12 (66)	
2	2 (6)	0 (0)	2 (11)	
3	1 (3)	0 (0)	1 (6)	
Femoral component loosening n (%)	1 (3)	0 (0)	1 (6)	1.000
Revision n (%)	1 (3)	0 (0)	1 (6)	1.000
VAS Mean ± SD	20.1 ± 20.6	18.3 ± 25.4	21.5 ± 16.9	0.235
Merle d'Aubigné-Postel Score Mean ± SD	16.8 ± 1.16	17.1 ± 0.83	16.6 ± 1.39	0.548

**Figure 2:**
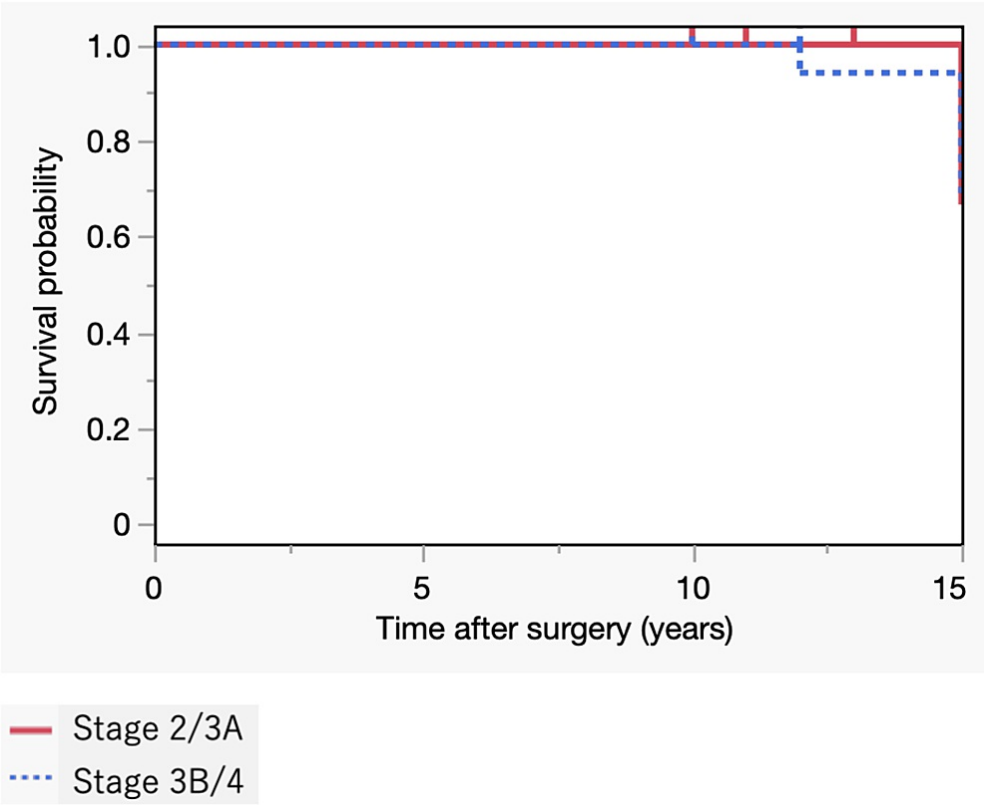
Kaplan–Meier Survival Curves with the endpoint of revision surgery (preoperative stage 2/3A vs. 3B/4 in new-generation BHA)

Preoperative stage progression was frequently observed in patients with more extensive necrosis (type C2). Only one (5%) patient in stage 3B/4 underwent revision 12 years after BHA due to the loosening of the femoral component and migration of the outer head. The Baker’s acetabular erosion grade was significantly higher in the stage 3B/4 group than in the stage 2/3A group (p<0.001). The Kaplan-Meier method was used to calculate the original prosthesis's retention probability. The 10-year survival rates were (16/16, 18/18) 100% in both groups (Figure 2).

The VAS and Merle d'Aubigné-Postel Score at the last visit were obtained in 11 (69%) hips of the 2/3A stage and 14 (82%) hips of the 3A/4 stage, excluding a revision case. No significant differences were found between the two groups in the VAS and Merle d'Aubigné-Postel Score (18.3±25.4 vs. 21.5±16.9, stage 2/3A vs. stage 3B/4, respectively, p=0.235 and 17.1±0.83 vs. 16.6±1.39, stage 2/3A vs. stage 3B/4, respectively, p=0.548).

## Discussion

In this study, we demonstrated that BHA for young patients with ONFH has excellent results over 15 years. Implant survival was better for the modern design than for first-generation BHA implants. Moreover, the preoperative stage influenced long-term survival significantly. There are several previous reports on BHA for ONFH cases without degeneration of the acetabulum, specifically for ONFH with Ficat classification or Association Research Circulation Osseous classification stage 3 or less (Table 4) [10,15-22].

**Table 4:** Summary of previous reports on mid/long-term results of BHA for ONFH BHA, bipolar hemiarthroplasty; ARCO: Association Research Circulation Osseous

Reference	Implant	Operation term	Mean follow-up (years)	Number of hips	Classification	Mean age (years)	Revision hip surgery	Survival rate (%)
Nagai et al., 2002 [17]	1st-generation BHA	1981–1984	14.2	12	Ficat 3	37	3	75
Muraki et al., 2008 [18]		1976–1995	13.7	16	Ficat 3	48.8	7	56
Hwang et al., 2012 [19]		1985–1993	20.3	55	Ficat 3	42.2	6	79
Current study (2024)		1984–1997	22.1	20	Ficat 2, 3, 4	41.8	7	65
Kim et al., 2012 [20]	New-generation BHA	1994–2005	7.9	115	Ficat 3	45.8	4	96.6
Gęgała et al., 2013 [21]		1998–2005	9.5	39	Ficat 3	45.3	3	92.3
Abe et al., 2017 [10]		1996–2014	7.6	79	Ficat 2, 3	53.5	3	96.3
Baba et al., 2020 [22]		1998–2010	12.8	62	ARCO 3	50.1	3	95.5
Current study (2024)		1998–2008	17.2	34	Ficat 2, 3, 4	45	1	97.1

Reports of surgeries performed before the mid-1990s demonstrated fair to poor outcomes, with revision rates ranging from 3% to 44% with mid-to-long-term follow-up. Conversely, reports after the late 1990s have shown relatively favorable mid-term outcomes, with 3%-8% revision rates. During the early phase, the suboptimal design of BHA implants and broad surgical indications to stage 4 ONFH might have led to unfavorable results due to implant migration, impingement, and osteolysis.

Implant-related factors include neck-rim impingement due to inadequate oscillation angles and third-body wear mode on the sliding surface due to polyethylene and metal debris generated by impingement [7]. To address these issues, the rim portion has been flattened to increase the oscillation angle in new-generation BHA implants [8,10]. Additionally, a locking ring was installed, and femoral components with highly polished necks, instead of those with a matte finish, were implanted to reduce excessive wear during impingement [23-25]. The porous coating of the femoral component has also improved [26]. Although there have been few direct comparisons of postoperative outcomes in BHA for different implant designs, our results indicated that the improvements in BHA implants have substantially impacted postoperative outcomes.

As for the indications for BHA, most reports of BHA for osteoarthritis with acetabulum cartilage change have shown poor long-term outcomes [27]. Kaku et al. [28] reported that the linear wear of the bearing surface after BHA was lower in patients with ONFH stage ≤3, in which a self-centering function in the bipolar cup works, than in those with osteoarthritis. In our study, it is possible that including patients with more advanced stages in the first-generation BHA group may have influenced the outcomes. When considering BHA for ONFH, the preoperative disease stage should be carefully assessed to ensure good long-term outcomes.

In a comparison between BHA and THA for ONFH, a few studies have concluded that BHA is superior to THA [6]. Other studies have reported that BHA for young patients had worse results than those of THA [29]. Although revision surgery might be required after initial surgery for ONFH in younger patients, regardless of BHA or THA, BHA without acetabular reaming likely preserves more bone stock on the acetabular side than that preserved by THA [10]. Additionally, the technically less demanding nature of revision is another advantage of BHA because the acetabulum is still intact [30]. However, for BHA, care should be taken, particularly in instances of bipolar head migration, as advanced migration might reduce bone stock on the acetabular side. Therefore, it is essential to follow up on patients regularly after surgery to determine the need for revision at the appropriate time. Furthermore, our results were compatible with those of earlier reports that BHA in cases of advanced-stage ONFH can lead to outer head migration and early revision. Considering the long-term results, we believe that THA should be the first choice for such patients. On the other hand, patients undergoing BHA with stage 2/3A only had grade 1 or less acetabular erosion, even after approximately 20 years of follow-up; this suggests that sufficient bone stock can be preserved in the long term and that BHA is highly advantageous for future revisions in these patients.

Although some reports state that the application of BHA for ONFH is limited [29], the results of our long-term analyses suggested that favorable outcomes can be achieved if new-generation BHA implants are used for early-stage ONFH. The advantages of BHA, such as the minimally invasive nature, low risk of dislocation, and ability to preserve acetabulum bone stock, may benefit young patients who are likely to require revision in the future.

This study had some limitations. First, it was a retrospective study involving few cases and it was conducted at a single center with no controls. Adjustment for confounding factors was limited owing to the small sample size. However, ONFH is a relatively rare condition, and among those who undergo arthroplasty, the number of patients suitable for long-term follow-up becomes limited. In the future, it is essential to conduct investigations with a large number of cases. Second, the first-generation BHA implant group had a significantly higher number of cases with advanced preoperative stages than that for the newer implant group, potentially skewing the results towards unfavorable outcomes. In other words, the effect of inappropriate surgical indications in the early stages could not be ruled out. Comparing the outcomes of past first-generation BHA and new-generation BHA, taking our results into consideration, it can be inferred that advancements in implant technology contribute to improved postoperative outcomes. Third, although all the implants were cementless new-generation BHA devices, various implant designs were used during the study. There is evidence that differences in implant construction and surfaces in the same generation can affect long-term survival [7]. Lastly, the patients in this study were all East-Asian. The frequency of femoral head necrosis and body size varies by race; therefore, the results of this study might not be generalizable to patients of other ethnicities. These issues should be addressed in future research.

## Conclusions

In conclusion, new-generation BHAs had significantly better implant survival for early-stage ONFH than that of first-generation BHAs. It is undeniable that THA is the main treatment option for advanced-stage ONFH. However, considering the benefits of BHA, which allows for long-term preservation of the acetabular bone compared to THA, as indicated by our research results, we believe that BHA is an acceptable treatment option for early-stage ONFH cases, particularly in younger patients who are at high risk of requiring revision surgery in the future.
